# Accuracy and reproducibility analysis of different reference axes for femoral prosthesis rotation alignment in TKA based on 3D CT femoral model

**DOI:** 10.1186/s12891-023-06781-4

**Published:** 2023-08-18

**Authors:** Kun Liu, Xuande Liu, Yujun Guan, Haotong Ma, Donglin Fu, Zongqing Fan

**Affiliations:** 1 Fuyang People’s Hospital, Anhui Medical University, NO.501 Sanqing Road, Yingzhou District, Fuyang, 236000 Anhui Province China; 2 China Railway Fuyang Hospital, Fuyang, 236000 Anhui Province China

**Keywords:** Three-dimensional CT, Posterior condylar angle, Femoral prosthesis rotation, Precision, Reproducibility

## Abstract

**Background:**

There are many reference axes to determine the rotational positioning of the femoral prosthesis in total knee arthroplasty (TKA), mainly including the surgical transepicondylar axis (sTEA), anatomical transepicondylar axis (aTEA), Whiteside line, and the posterior condylar line (PCL), etc., but there is still no definite conclusion on which is the most accurate reference axis.

**Objective:**

To explore the reproducibility of each reference axis of femoral external osteotomy based on the 3D CT femoral model, compare the deviation of the simulated femoral prosthesis rotation alignment, positioned based on each reference axis, with the gold standard sTEA, and analyze the accuracy of each reference axis.

**Methods:**

The imaging data of 120 patients with knee osteoarthritis who underwent a 3D CT examination of the knee in our hospital from June 2018 to December 2021 were retrospectively collected. The 3D model of the femur was established by Mimics software. The line relative to PCL externally rotated 3° (PCL + 3°), aTEA, and the vertical line of the Whiteside line were constructed and compared with the gold standard sTEA. Intra-observer, as well as inter-observer reproducibility analysis, was performed by the intra-group correlation coefficient (ICC) and Bland-Altman method.

**Results:**

The angle ∠WS, between the vertical line of Whiteside and sTEA, was 2.54 ± 2.30°, with an outlier of 54.2%; the angle ∠aTEA, between aTEA and sTEA, was 4.21 ± 1.01°, with an outlier of 99.1%; the angle ∠PCL, between PCL + 3° external rotation and sTEA, was 0.50 ± 1.06°, with the highest accuracy and an outlier of 5.8%, and the differences among all three were statistically significant, *P* < 0.05. The intra-observer ICC values of ∠WS, ∠aTEA, and ∠PCL were 0.975 (0.964–0.982), 0.926 (0.896–0.948), and 0.924(0.892,0.946), respectively, and the reproducibility levels were excellent; the inter-observer ICC values of ∠WS, ∠aTEA, and ∠PCL were 0.968(0.955–0.978), 0.906 (0.868–0.934) and 0.970 (0.957,0.979), respectively, with excellent reproducibility levels; Bland-Altman plots suggested that the scatter points of intra-observer and inter-observer measurement differences more than 95% were within the limits of agreement.

**Conclusion:**

The reference axis for locating the distal femoral external rotation osteotomy based on the 3D CT femoral model has good reproducibility. The PCL is easy to operate, has the highest precision, and the lowest outliers among the reference axes is therefore recommended.

## Introduction

Osteoarthritis of the knee occurs in the elderly, and the advanced stage can lead to severe joint pain and deformity, which can seriously affect the life of patients. Total knee arthroplasty (TKA) is the most effective treatment for advanced knee osteoarthritis, as it can effectively relieve knee pain, improve knee function, and enhance patients’ quality of life [1]. Poor positioning of the rotational alignment of the femoral prosthesis in TKA can lead to a series of complications, including anterior knee pain, limited knee motion, poor patellar trajectory, and prosthesis loosening [2]. Therefore, how to accurately position the femoral prosthesis rotation alignment intraoperatively has been a long-standing concern for orthopedic surgeons.

The surgical transepicondylar axis (sTEA), first defined by Berger et al., is the line connecting the most prominent point of the lateral epicondyle of the femur, with the medial epicondylar sulcus [3]. In knee flexion and extension activities, the sTEA has been shown to be the axis with high overlap with the functional axis of knee flexion and extension, and it is most likely to create a balanced flexion gap when positioning the femoral prosthesis rotation with reference to the sTEA intraoperatively [4, 5]. In addition, the application of surgical computer navigation techniques has further confirmed the consistency of the sTEA with the functional axis of knee motion, and relevant anatomical and biomechanical analyses have revealed no significant differences between the two [6]. Therefore sTEA is considered the gold standard for determining the rotational alignment of the femoral prosthesis. However, intraoperative localization of the sTEA can be difficult because the sulcus of the medial epicondyle is often covered by dense soft tissue such as the deep fibers of the medial collateral ligament [7], which may lead to varying degrees of error based on the visual observation and touch localization [8].

Therefore, alternative reference axes such as the anatomical transepicondylar axis (aTEA), Whiteside line, and posterior condylar line (PCL) have been reported based on the measured resection technique for external rotation osteotomy of the femur. However, which reference axis deviates the least from the sTEA is still controversial [9, 10]. In addition, most of the previous studies were based on two-dimensional CT images, and the accuracy of the reference axes was evaluated at the most obvious CT plane of the studied anatomical axes [11]. In practice, the sTEA and the reference axes are most likely not in the same CT horizontal section, so there is a certain error in measuring in the same CT plane. However, there is a lack of studies based on three-dimensional measurements and large sample sizes to comprehensively compare the accuracy of intraoperative commonly used reference axes for positioning the femoral prosthesis rotation alignment. Therefore, the purpose of this study is to reconstruct a 3D model of the femur based on CT scan data of the knee using Mimics software, precisely locate each reference axis and project them onto the same plane, compare the deviation of the simulated rotational alignment of the femoral prosthesis positioned based on each reference axis to the gold standard sTEA, and analyze the accuracy as well as the reproducibility of each reference axis.

## Materials and methods

### Research materials

Three-dimensional CT of the knee and full-length anteroposterior X-ray images of both lower limbs were retrospectively analyzed of 186 patients from June 2018 to December 2021 at Fuyang People’s Hospital, affiliated with Anhui Medical University. Finally, 120 cases were enrolled according to the inclusion and exclusion criteria, including 45 males and 75 females; ages ranged from 26 to 77 years, with an average of 50.18 ± 10.92 years. The study was approved by the Ethics Committee of Fuyang People’s Hospital (IRB: [2022]79) and exempted from the requirement for informed consent. The study was conducted in accordance with the ethical norms and guidelines established by this committee.

**Fig. 1:**
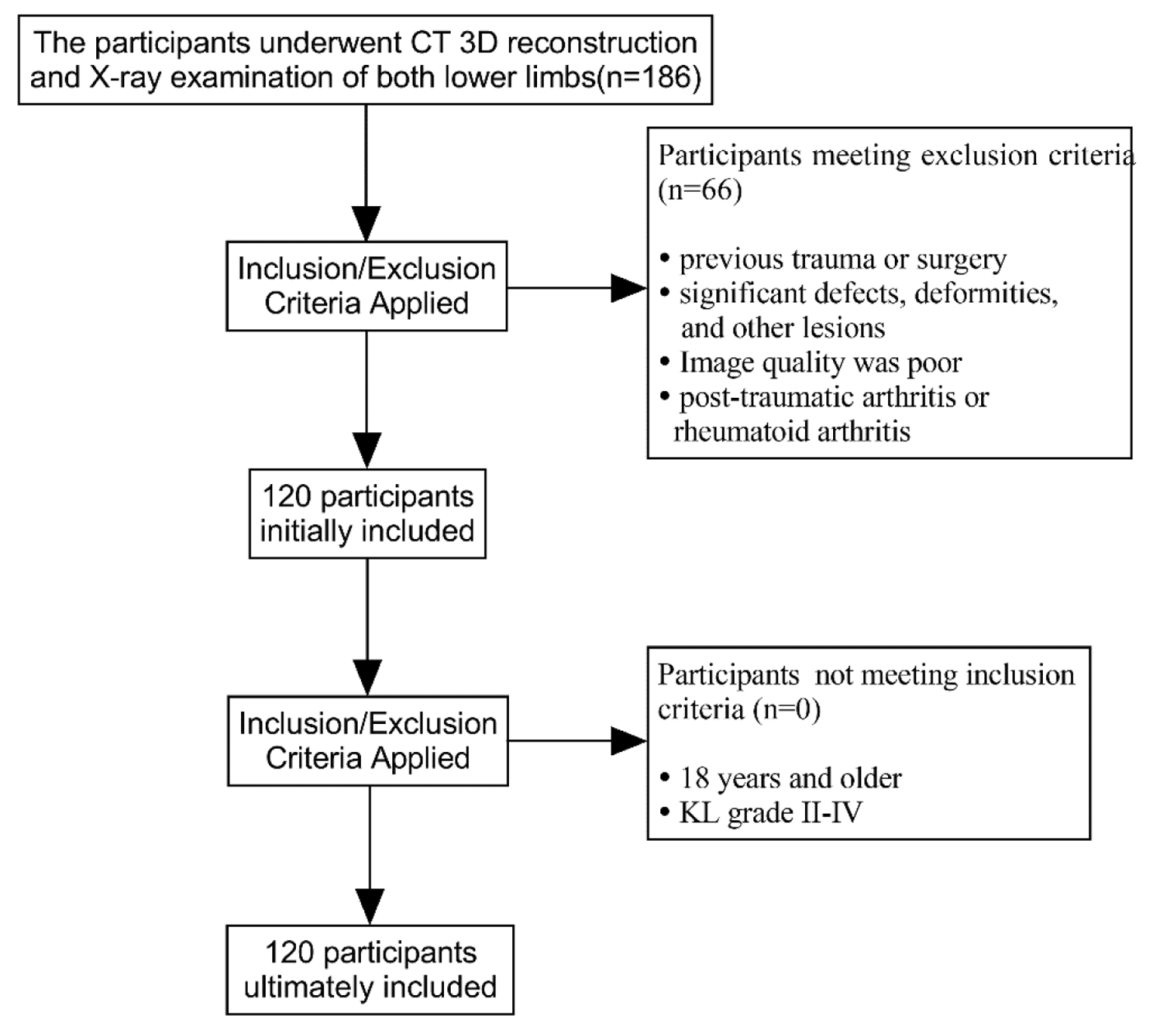
The participant selection process

Inclusion criteria: patients aged 18 years and older; simultaneous 3D CT of the knee and full-length anteroposterior X-ray images of both lower limbs; osteoarthritis of the knee KL (Kellgren-Lawrence, KL) grade II-IV. Exclusion criteria: measurement of the distal femur was affected by previous trauma or surgery; the presence of significant defects, deformities, and other lesions in the femur that affect the measurement; CT scan was not standardized, and image quality was poor; patients with post-traumatic arthritis or rheumatoid arthritis. The screening process is shown in Fig. 1.

### Imaging examination

#### 3D CT examination of the knee

The patient was supine on the CT examination table with both lower limbs rotated to neutrality, and the patella and toes pointed upward. Scanning parameters: tube voltage 120 kV, tube current 150 mA, display field 27.3*27.3 cm, exposure time 1000 ms. The scanning layer is perpendicular to the mechanical axis of the femur, and the layer thickness is 1 mm. The CT image data were stored in DICOM format. Modeling was performed using Mimics Research 19.0 software (Materialize, Belgium), and parameters were measured using the software’s own measurement tool.

#### Weight-bearing and full-length anteroposterior X-ray examination of both lower limbs

The patient was examined using a digital X-ray imaging camera (GE Healthcare, US) with the patient standing upright on the camera frame with the back pressed against the frame and hands hanging naturally, knees as straight as possible, feet shoulder-width apart, internally rotated about 15°, distance from the X-ray machine flat detector 1.6-1.8 m, keeping the body still, showing a field of 37.0*105.0 cm, the head of the fibula on the X-ray film overlap with the tibia by about 1/3, and the patella points directly anterior.

### Determination of femoral valgus angle

**Fig. 2:**
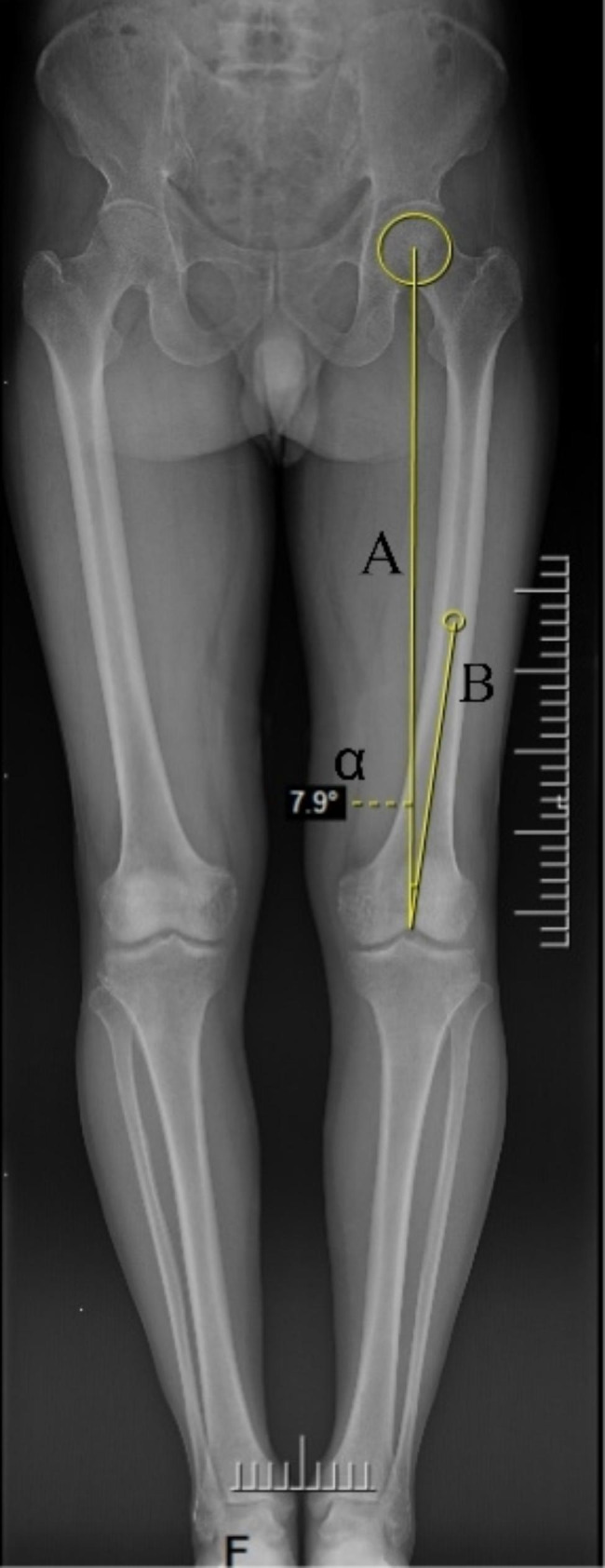
Measurement of femoral valgus angle in full-length radiographs of both lower limbs. Note: **A**: mechanical axis of the femur. **B**: anatomical axis of the femur. α: femoral valgus angle

The patient’s full-length radiographs of both lower limbs were shown in the Pacs image viewing system (Infinitt Healthcare, Korea), and a circle was drawn to fit the edge of the femoral head, with the center of the circle being the center of the hip joint; the center of the most distal cortex of the intercondylar notch of the femur was selected as the center of the knee joint, and the line connecting the two centers was the mechanical axis (axis A) of the femur; the medullary canal of the femur was fitted by drawing a circle 20 cm above the knee joint line [12], and the line connecting the center of this circle to the center of the knee joint was the anatomic axis (axis B) of the femur [13]. The angle between the mechanical axis and the anatomical axis of the femur is known as the femoral valgus angle, denoted as α., noted as α. The valgus angle α in this case is 7.9°. Figure 2.

### Modeling of the femur and determination of the measurement plane

#### Construction of the femoral 3D model

**Fig. 3:**
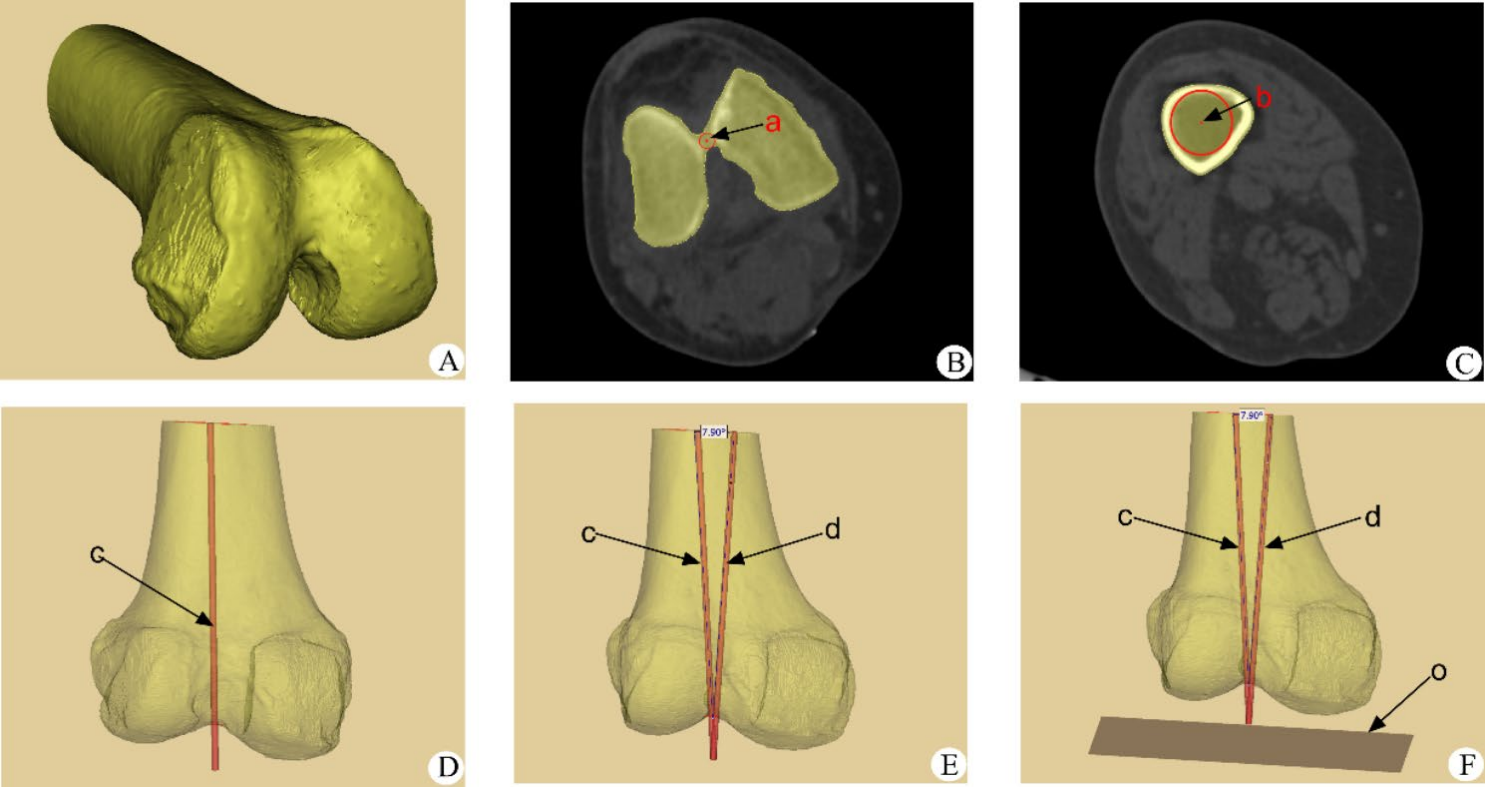
Construction of the 3D model of the femur and determination of the transverse section of the mechanical axis of the femur. Note: Fig. 2A: 3D model of the reconstructed femur in Mimics software. Fig **B** ~ **D**: positioning of the anatomical axis of the femur a: most vertex of the intercondylar notch. b: center of the medullary canal of the femoral shaft. c: anatomical axis of the femur. Fig **E**: locating the femoral mechanical axis by 7.9° varus relative to the anatomical axis. c: the anatomical axis of the femur. d: the femoral mechanical axis. Fig **F**: Determination of the transverse section of the femoral mechanical axis. Gray plane o: transverse section of the femoral mechanical axis

The stored CT image data in DICOM format were imported into Mimics software, and the 3D model of the distal femur was reconstructed by marking, creating contour lines, and filling the cavity, as shown in Fig. 3.

#### Determination of the measurement plane for measuring the deviation of the rotational alignment of the femoral component

We draw a circle in the cross section of the femoral shaft to make it fit the medullary canal of the femur to the maximum, and the center of the circle is the center of the medullary canal. At the same time, find the last cross section of the distal femur containing the cortex of the vertex of the intercondylar notch of the femur, and draw a circle here to make the front and rear edges of the circle tangent to the anterior and posterior cortex of the vertex. The center of the circle is the apex of the intercondylar notch. At the same time, the line between the two centers of the two circles is the anatomical axis of the femur. Then the angle α(7.9°) between the anatomical axis and mechanical axis measured in the full-length radiographs of both lower limbs was used to determine the mechanical axis of the femur model, and finally, the vertical plane of the mechanical axis was determined, which was defined as the transverse section “o” [14]. Each reference axis was projected onto the transverse plane “o”, and then the deviation between the rotational alignment of the femoral component simulated based on each reference axis and the sTEA was measured. Figure 3.

### Positioning of anatomical landmarks of distal femur and simulated femoral prosthesis rotation alignment

**Fig. 4:**
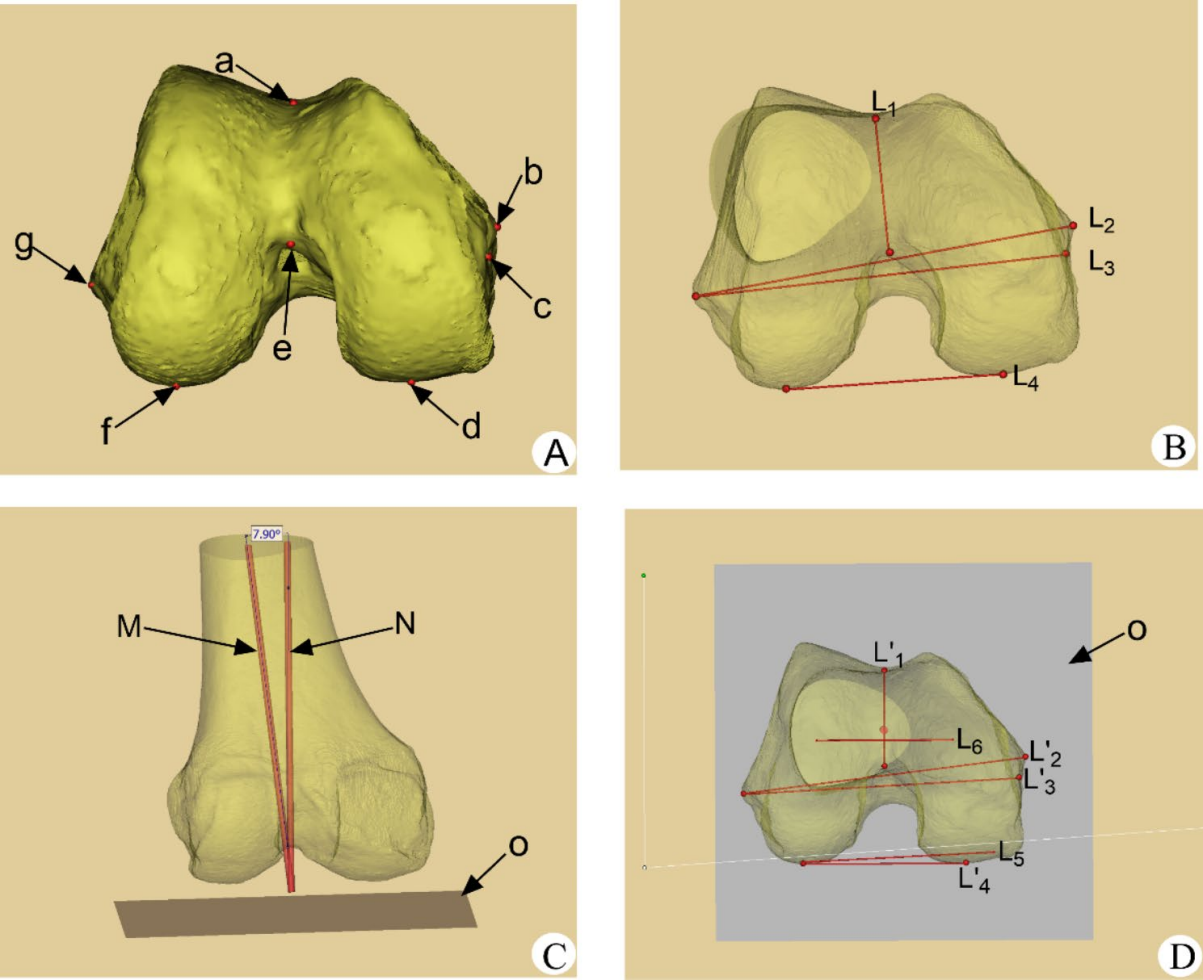
Positioning of anatomic landmarks of the distal femur and the simulated femoral component rotational alignment in transverse section. Note: Fig. **A**: The positioning of anatomical landmarks. a: the deepest point of femoral trochlear groove. b: the most prominent point of the medial epicondyle. c: the medial epicondylar sulcus. d: the lowest point of the medial posterior condyle. e: the deepest point of femoral posterior intercondylar notch. f: the lowest point of the lateral posterior condyle. g: the most prominent point of the lateral epicondyle. Fig. **B**: Positioning of the reference axis of the rotational osteotomy of the femur. L_1_: Whiteside line. L_2_:aTEA. L_3_:sTEA. L_4_:PCL Fig. **C**: Positioning of the transverse section of femoral mechanical axis. Gray plane "o": the transverse section plane. M: the anatomical axis of the femur. N: the mechanical axis of the femur. Fig. **D**: Positioning of the simulated rotation alignment of the femoral prosthesis in transverse section “o”. L’_1_: projection of L_1_ on the transverse section. L’_2_: projection of L_2_ on the transverse section. L’_3_: projection of L_3_ on the transverse section L’_4_: projection of L_4_ on the transverse section. L_5_: the external rotation of L’_4_ by 3°. L_6_: the vertical line of L’_1_. Gray plane “o”: the transverse section plane

In the 3D model of the distal femur reconstructed by Mimics software, the bony landmark points were marked following previously published studies, and then each reference axis was determined. sTEA is the line between the most prominent point of the lateral epicondyle and the sulcus of the medial epicondyle [7]; aTEA is the line between the most prominent point of the medial and lateral epicondyles [7]; Whiteside line, also known as the anterior-posterior axis (AP axis) is the line connecting the deepest points of the anterior and posterior intercondylar notch of the femur when the femur is viewed in the axial position [15]; PCL is the line connecting the lowest points of the medial and lateral posterior condyles of the femur. Finally, all the reference axes are projected into the transverse section “o”. The rotational alignment of the femoral component simulated based on the Whiteside line is the perpendicular line to the Whiteside line in the transverse section “o”; The femoral component rotational alignment simulated based on the aTEA is the line that corresponds to the aTEA in the plane “o”; The femoral component rotational alignment simulated based on the PCL reference is the line that corresponds to the PCL + 3° external rotation in the plane “o”. Figure 4.

### Measurement indexes

**Fig. 5:**
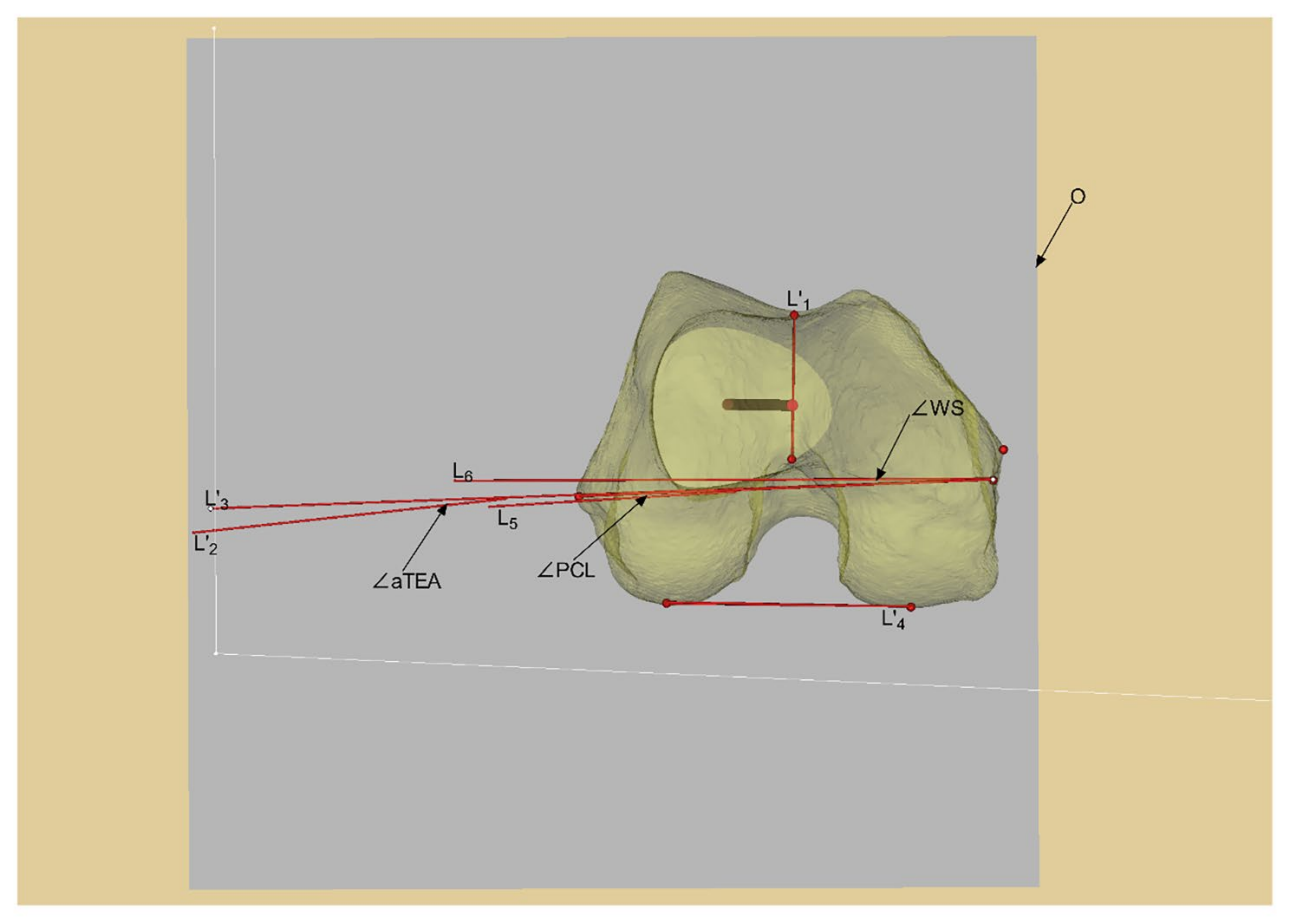
Measurement of the angle between the simulated femoral prosthesis rotation alignment positioned by reference axis and the sTEA on the transverse section of the femoral mechanical axis. Note: L’_1_: projection of whiteside line on the transverse section L’_2_: projection of aTEA on the transverse section. L’_3_: projection of sTEA on the transverse section. L’_4_: projection of PCL on the transverse section. L_5_: the external rotation of L’_4_ by 3° on the transverse section. L_6_: the vertical line of L’_1_ on the transverse section. Gray plane o: the transverse section plane

All reference axes and sTEA were projected onto the transverse section “o”, and the measurement of the angle between the simulated femoral prosthesis rotationl alignment positioned by each reference axis and sTEA was performed on the transverse section “o”. The deviation of the simulated rotational alignment of the femoral component based on the Whiteside line reference is the angle between the perpendicular line to the Whiteside line and the sTEA, denoted as ∠WS; The deviation of the simulated rotational alignment of the femoral component based on the aTEA reference is the angle between the aTEA and the sTEA, denoted as ∠aTEA; The deviation of the simulated rotational alignment of the femoral component based on the PCL reference is the angle between the PCL + 3° external rotation and the sTEA, denoted as ∠PCL. The angles of the deviation of the simulated femoral prosthesis rotation alignment were recorded as positive when it was externally rotated to the sTEA and negative when it was internally rotated. These angles were measured and compared, and deviations of >±2° [16] were categorized as outliers to evaluate the data of this study. Measurements were retaken two weeks later [17]by the same measurer and another measurer to assess intraobserver and interobserver reproducibility. All measurers who performed the measurements were well-trained for the relevant measures, Fig. 5.

### Statistical analysis

SPSS 25.0 software was used for analysis. Quantitative data conforming to normal distribution or approximately normal distribution were described by $\stackrel{-}{x}$±$S$. The overall comparison between groups of each reference axis was performed by ANOVA, Welch test was used when the variance was not equal, and the LSD method was used for pairwise comparison between groups. The intraclass correlation coefficient (ICC) and Bland-Altman method were used to assess intra-observer and inter-observer repeatability. ICC evaluation criteria ICC < 0.4 indicated poor consistency, 0.40 to 0.75 indicated good consistency, and > 0.75 showed high consistency [18]. Evaluation criteria for Bland Altman analysis: at least 95% of the differences between measurements lie within the 95% limits of consistency, i.e., the limits of consistency contain more than 95% scatter points [19]. *P* < 0.05 was considered statistically significant.

### Results

### Consistency test for deviations between femoral prosthesis rotational alignment positioned by three reference axes and sTEA

#### Intra-observer consistency

The ICC values of ∠WS, ∠aTEA, and ∠PCL measured twice by the same measurer were 0.975 (0.964–0.982), 0.926 (0.896–0.948), and 0.924 (0.892,0.946), respectively, all with high levels of consistency, as shown in Table 1.

**Fig. 6:**
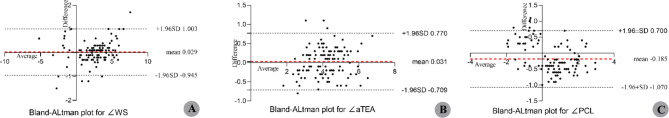
Bland-Altman plot of the angle between the femoral prosthesis rotational alignment and sTEA measured twice by the same measurer. Note: ∠WS: angle between the perpendicular line of Whiteside line and sTEA. ∠aTEA: angle between aTEA and sTEA. ∠PCL: angle between PCL + 3° external rotation and sTEA

The scatter of the difference between the two measurements of ∠WS, ∠aTEA, and ∠PCL within the observer all lie within the consistency limits by more than 95%, Fig. 6.

**Table 1:** ICC values of the intra-observer angle between the femoral component rotational alignment and the sTEA ($\stackrel{-}{x}$±$S$)

item	∠WS	∠aTEA	∠PCL
First measurement	2.54 ± 2.30	4.21 ± 1.01	0.50 ± 1.06
Second measurement	2.51 ± 2.14	4.17 ± 0.95	0.69 ± 1.25
ICC(95%CI)	0.975(0.964 ~ 0.982)	0.926(0.896 ~ 0.948)	0.924(0.892,0.946)

Note: ∠WS: angle between the perpendicular line of Whiteside line and sTEA. ∠aTEA: angle between aTEA and sTEA. ∠PCL: angle between PCL + 3° external rotation and sTEA

### Inter-observer consistency

The ICC values of ∠WS, ∠aTEA, and ∠PCL measured by different observers were 0.968 (0.955–0.978), 0.906 (0.868–0.934), and 0.970 (0.957,0.979), respectively, all with high levels of agreement, Table 2.

**Fig. 7:**
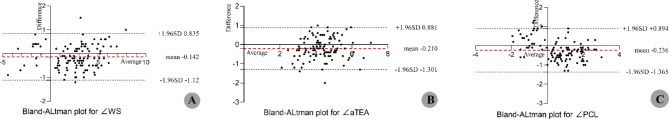
Bland-Altman plot of the angle between the femoral prosthesis rotational alignment and sTEA measured twice by the different measurers. Note: ∠WS: angle between the perpendicular line of Whiteside line and sTEA. ∠aTEA: angle between aTEA and sTEA. ∠PCL: angle between PCL + 3° external rotation and sTEA

The scatter of the difference between the two measurements of ∠WS, ∠aTEA, and ∠PCL within the observer all lie within the consistency limits by more than 95%, Fig. 7.

**Table 2:** ICC values of the inter-observer angle between the femoral component rotational alignment and the sTEA ($\stackrel{-}{x}$±$S$)

Item	∠WS	∠aTEA	∠PCL
Observer A	2.51 ± 2.14	4.17 ± 0.95	0.69 ± 1.25
Observer B	2.68 ± 2.23	4.42 ± 0.94	0.74 ± 1.30
ICC(95%CI)	0.968(0.955 ~ 0.978)	0.906(0.868 ~ 0.934)	0.970(0.957,0.979)

Note: ∠WS: angle between the perpendicular line of Whiteside line and sTEA. ∠aTEA: angle between aTEA and sTEA. ∠PCL: angle between PCL + 3° external rotation and sTEA

### Comparison of the accuracy of femoral prosthesis rotational alignment positioned by the three reference axes

**Fig. 8:**
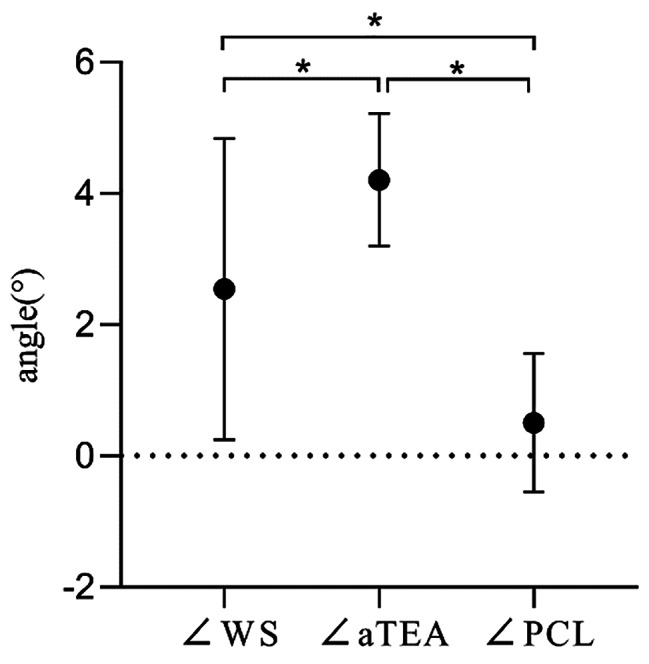
Comparison of the deviations of the femoral component rotational alignment positioned by the three reference axes with the sTEA. Note: ∠WS: angle between the perpendicular line of Whiteside line and sTEA. ∠aTEA: angle between aTEA and sTEA ∠PCL: angle between PCL + 3° external rotation and sTEA. *difference was statistically significant

The angles of each femoral prosthesis rotational alignment with sTEA, i.e., ∠WS, ∠aTEA, and ∠PCL were 2.54 ± 2.30°, 4.21 ± 1.01°, and 0.50 ± 1.06°, respectively. PCL + 3° external rotation with sTEA error was smaller relative to the Whiteside line as well as aTEA, and the difference was statistically significant, *P* < 0.05, Fig. 8.

### Distribution of outliers of deviation from the gold standard sTEA for the femoral prosthesis rotational alignment positioned by the three reference axes

**Fig. 9:**
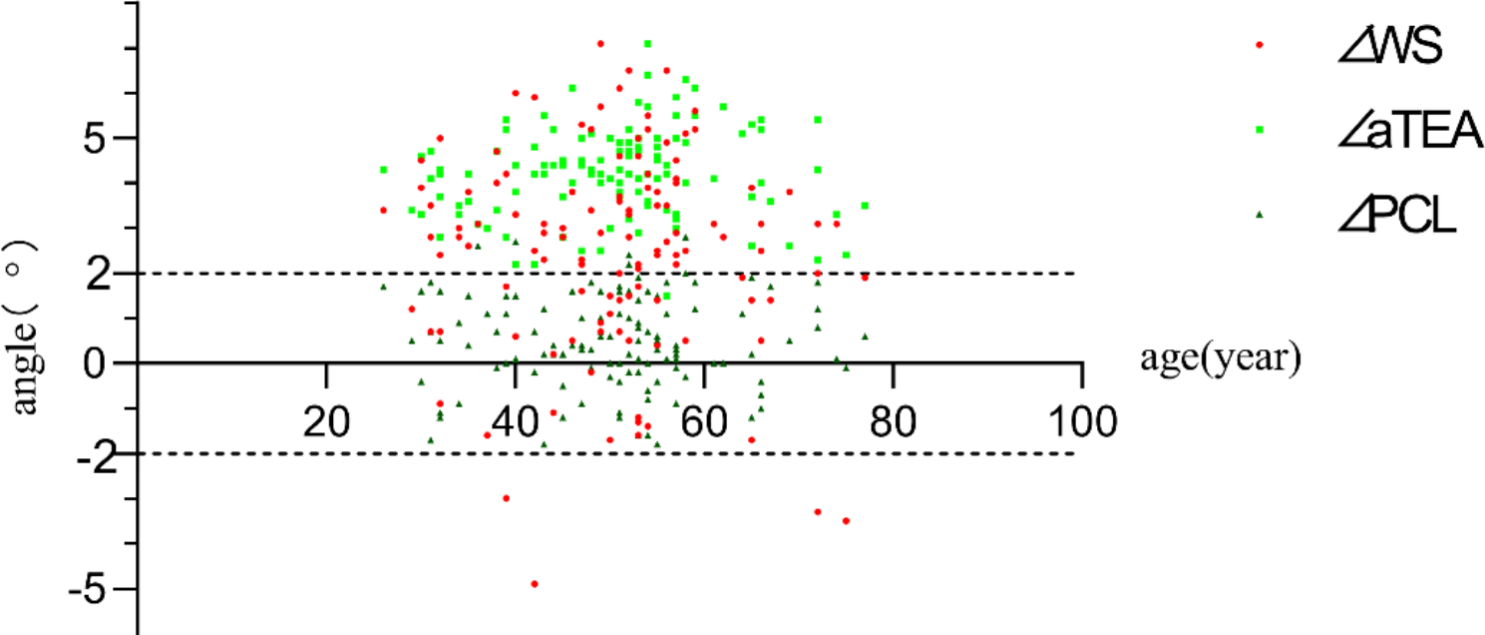
Distribution of outliers of the difference between the rotational the three femoral rotational prostheses and the gold standard sTEA. Note: ±2° is the error boundary ∠WS: angle between the perpendicular line of Whiteside line and sTEA. ∠aTEA: angle between aTEA and sTEA. ∠PCL: angle between PCL + 3° external rotation and sTEA

The outliers for the angle of rotation alignment of the femoral prosthesis positioned by each reference axis to the sTEA are 65 (54.2%) (∠WS), 119 (99.1%)(∠aTEA) and 7 (5.8%) (∠PCL), respectively, and the scatter distribution is shown in Fig. 9.

## Discussion

Poor rotation of the femoral prosthesis can lead to a series of complications [20]. The choice of the most optimal reference axis to position the femoral rotational alignment is still controversial [21]. Considering the limitations of localizing and comparing the precision of each reference axis in the same plane of 2D CT slice and the fact that no study to date has proposed an ideal method for localizing the reference axis of the femoral prosthesis rotational alignment using 2D CT. In this study, based on 3D CT data of the knee, a 3Dmodel of the femur was reconstructed using Mimics software to achieve accurate positioning of various reference axes, which were then projected onto the same transverse section, and the accuracy and reliability of the reference aTEA, whiteside line, and PCL in determining the femoral prosthesis rotational alignment were compared comprehensively based on a relatively large sample size.

In this study, each reference axis was located in the 3D model of the femur. Its deviation from sTEA was measured, excluding the influence of soft tissue coverage and intraoperative visual field limitation on positioning [7], allowing for relatively accurate positioning. The ICC values within and between observers are greater than 0.75, which can be considered good repeatability according to the evaluation criteria. At the same time, combined with the Bland-Altman plots, the scatter points of the difference of the angle between each reference axis and sTEA within and between observers are more than 95% within the consistency limit, which indicates that the positioning and measurement of the deviation between each reference axis and sTEA in the 3D model with robust repeatability. Our results are consistent with the results reported by Lei et al. [10], who positioned and measured the deviation of the Whiteside line and the PCL positioned femoral prosthesis rotational alignment from sTEA in a reconstructed 3D CT model of the femur, with intra- and inter-observer ICC > 0.9, with good reproducibility. The results of the study conducted by Robertson et al. [22] also indicated that compared to 2D CT, the accuracy and repeatability of locating reference axes were higher in 3D CT models. However, many studies [10, 23] have performed CT scans of the entire femur to locate the mechanical axis of the femur in the 3D model, which can increase the exposure time to radiation and additional financial burden. Compared to previous studies, the present study only utilized 3D CT data of the knee joint and combined it with the femoral valgus angle in full-length films to locate the transverse section of the femoral mechanical axis, ensuring accurate measurement of all indicators while also considering patient safety and economy.

In TKA, the measured resection technique mainly uses distal femoral anatomical landmarks to locate the distal femoral rotational osteotomy line. The primary reference axes commonly used during operation are aTEA, sTEA, PCL and the Whiteside line [24]. The sTEA has been widely accepted as the gold standard for rotational positioning of the femoral prosthesis [3, 7, 25].

Earlier studies have shown that the Whiteside line is stable to individual differences in femoral morphology and is not affected by gender or ethnicity [15]. Because the Whiteside line is based on the non-weight-bearing area of the distal femoral surface, it is relatively stable to changes in cartilage thickness due to knee degeneration. However, the deviation of the femoral prosthesis rotational alignment positioned by the Whiteside line from the sTEA in this study was 2.54 ± 2.30°, an outlier of 54.2%, indicating that the Whiteside line is not the ideal reference axis, which is in line with the findings of most previous studies [26, 27]. To analyze the reason, it may be due to more osteophyte hyperplasia in some patients, and the femoral intercondylar fossa is closed by osteophytes. Before positioning the Whiteside line, it is often necessary to remove the osteophytes in the intercondylar fossa, which may change the shape of the intercondylar fossa, resulting in inaccurate positioning of the Whiteside line [28, 29]. Nagamine et al. [27] found that the vertical line of the Whiteside line was 1.4 ± 3.3 ° internal rotation relative to sTEA through CT scanning of 84 knee joints and concluded that the reliability of determining the femoral prosthesis rotational alignment with reference to Whiteside line was lower than that of PCL when performing TKA for medial tibiofemoral arthritis.

The transepicondylar axis (TEA) is the line connecting the most prominent points of the medial and lateral epicondyles of the femur. It can be divided into the sTEA (the line connecting the most prominent points of the lateral epicondyle and medial epicondyle sulcus of the femur) and the aTEA (the line connecting the most prominent points of the lateral and medial epicondyle of the femur). Compared to the Whiteside line and sTEA, aTEA is relatively easy to position intraoperatively. The deviation of the femoral prosthesis rotational alignment with reference to aTEA from sTEA in this study was 4.21 ± 1.01°, an outlier of 99.1%, indicating that intraoperative parallel aTEA positioning of the external rotation osteotomy line of the femur is subject to significant error. This is similar to the findings of Jabalameli et al. [30], who performed CT scans of the knee in 108 patients with osteoarthritis of the knee and showed that aTEA showed 4.3° of internal rotation compared to sTEA. In addition, the results of this study found that the variability of aTEA was the smallest among the three (SD = 1.01°), which suggests that the deviation between aTEA and sTEA is more constant, which can be used to evaluate the femoral prosthesis rotation concerning aTEA when sTEA cannot be accurately identified.

PCL is easily identified during operation and is a relatively reliable reference axis. Many total knee replacement instruments currently use PCL + 3 ° external rotation to determine the femoral prosthesis rotational alignment. This value is based on the angle between PCL and sTEA measured by Berger et al. on the cadaver specimen; the posterior condylar angle (PCA) is 3.5 ± 1.2 °, which can be approximately parallel to sTEA with the conventional 3 ° external rotation osteotomy relative to PCL [7]. In addition, the proximal tibia physiologically varus articular surface was resected perpendicular to the tibial mechanical axis during osteotomy. An externally rotated 3 ° osteotomy would also be beneficial to create a stable flexion gap and maintain the consistency of tibiofemoral kinematics [31]. Our results indicate that PCL + 3 ° external rotation is closest to the physiological axis of knee flexion-extension movements, sTEA, with a mean difference of 0.5 ° and an outlier of 5.8%, which is significantly better than aTEA and Whiteside line. This is consistent with the findings of Jang et al. [9], who reconstructed 2128 femoral models and found a minor deviation from sTEA relative to the PCL + 3° external rotation osteotomy with an average of 0.60° and concluded that the PCL was the most accurate compared to other reference axes. But their included were all normal femora, and the included femora in this study were all KL grade II and above, so this study was closer to clinical reality, and the conclusions were more reliable. However, many studies that consider performing routine external rotation 3° osteotomy on patients do not take into account the anatomical variation of the distal femur [22, 32], the asymmetric wear, varus deformity, etc., which can make PCA individualized [33], and routine external rotation 3° osteotomy is not accurate. In a cadaveric study, Mantass et al. [34] reported that the sTEA relative to PCL external rotation angle ranged from − 1° to 7°, with a mean external rotation of 5°. Yip et al. [35] found a mean PCA of 5.8° in women and 5.1° in men by measuring 82 femoral specimens. Griffin et al. [36] used MRI to measure PCA in 104 knees. The results showed an increase in PCA with age. They considered that this was associated with wear of the posterior lateral femoral cartilage, leading to an increase in PCA at the time of measurement. The reasons for this may be related to differences in the anatomy of the distal femur in different ethnic groups and people with different characteristics [35, 37], in addition to the fact that different measurement methods may have an impact on the results [22]. In our opinion, although the deviation of PCL + 3° external rotation from sTEA has clear outliers, it is accurate in most cases. In addition, many studies with larger sample sizes confirm that the deviation of PCL + 3° external rotation from sTEA is more accurate relative to other reference axes [9, 27] and that the PCL is readily available intraoperatively and more reproducible, so we believe that reference to the PCL is still a reliable method for determining the femoral prosthesis rotational alignment when the medial and lateral posterior condyles are not severely disrupted.

The strength of this study was that the three-dimensional model of the distal femur was reconstructed using Mimics software, precisely positioned, and the deviation of each reference axis from the sTEA was measured and compared. In addition, some studies do not consider the effect of osteoarthritis on the anatomical structure of the knee [9]; all included in our study were knees with osteoarthritis KL grading, grade II or above, which is closer to clinical reality and more instructive for clinical practice.

However, this study also has shortcomings. Firstly, the reference axes analyzed in our study were only those commonly used intraoperatively; other reference axes were not included in the analysis; secondly, the reconstructed 3D model did not include cartilage structures and did not consider the influence of factors such as race, gender, and age on the anatomy of the distal femur; thirdly, the exclusion criteria may affect the generalizability of this study, but they also ensure the reliability and accuracy of the results; finally, our study did not compare the deviation of the rotational alignments of the femoral prosthesis positioned by each reference axis postoperatively, which could further justify our conclusions. The results of this study need to be further validated by multicenter clinical studies with large sample size. In the future, studies on the accuracy of the reference axis of the femoral prosthesis rotational alignment in TKA still need to use more precise methods and consider the influence of demographic characteristics and other factors on the anatomy of the femur.

### Conclusions

Femoral component rotational alignment has an essential impact on the prognosis of TKA, but a consensus reference axis with high precision has not been reached. The deviations of the femoral component rotational alignment positioned by each reference axis from the sTEA could be accurately and reliably positioned on the reconstructed 3D model of the distal femur, and the PCL + 3 ° external rotation was still closest to the physiological axis of knee flexion and extension.

## Data Availability

On reasonable request, the datasets generated and analyzed during the current study are available from the corresponding author.
